# Increased expression of interleukin-6 predicts poor response to chemoradiotherapy and unfavorable prognosis in oral squamous cell carcinoma

**DOI:** 10.3892/or.2015.3838

**Published:** 2015-03-06

**Authors:** TEPPEI JINNO, SHINTARO KAWANO, YASUYUKI MARUSE, RYOTA MATSUBARA, YUICHI GOTO, TAIKI SAKAMOTO, YUMA HASHIGUCHI, NAOKI KANEKO, HIDEAKI TANAKA, RYOJI KITAMURA, TAKESHI TOYOSHIMA, AKIKO JINNO, MASAFUMI MORIYAMA, KAZUNARI OOBU, TAMOTSU KIYOSHIMA, SEIJI NAKAMURA

**Affiliations:** 1 Section of Oral and Maxillofacial Oncology, Kyushu University, 3-1-1 Maidashi, Higashi-ku, Fukuoka 812-8582, Japan; 2 Laboratory of Oral Pathology, Division of Maxillofacial Diagnostic and Surgical Sciences, Faculty of Dental Science, Kyushu University, 3-1-1 Maidashi, Higashi-ku, Fukuoka 812-8582, Japan

**Keywords:** interleukin-6, oral squamous cell carcinoma, p-STAT3, chemoradiotherapy, chemoresistance, prognosis, tumor response, inflammation

## Abstract

Recent studies have revealed that cancer cells are exacerbated by chronic inflammation. The present study examined the immunohistochemical expression for interleukin-6 (IL-6), a pleiotropic inflammatory cytokine, in oral squamous cell carcinoma (OSCC) to elucidate the association of IL-6 expression with tumor progression, chemoresistance and prognosis. Seventy-eight patients with primary OSCC were analyzed by immunohistochemical staining for IL-6. These labeling indexes (LIs) were calculated and evaluated in association with the clinicopathologic characteristics and prognosis in the OSCC patients. The patients were divided into three groups as follows: negative group = LI <5%; low IL-6 group = 5% ≤ LI <30%; high IL-6 group = LI ≥30%. The patient numbers of the negative, low and high expression groups were 24, 22 and 32, respectively. In the high IL-6 expression group, IL-6 receptor (IL-6R), phospho-signal tranducer and activator of transcription 3 (p-STAT3) were also detected in almost all the cancer cells. The prevalence of the cervical lymph node or the distant metastasis in the high expression group was significantly higher than those in the negative and low expression groups. Furthermore, the high expression group had a significantly poorer tumor response to the preoperative chemoradiotherapy and a more unfavourable prognosis than the negative and the low expression groups. Interestingly, IL-6, IL-6R and p-STAT3 were expressed in the residual cancer cells of all the patients in the high expression group with poor response to chemoradiotherapy. These results suggested that IL-6 signaling possibly is involved in the progression and treatment-resistance of OSCC and IL-6 expression in cancer cells could be a useful predictive factor of poor response to chemoradiotherapy and unfavorable prognosis.

## Introduction

Oral squamous cell carcinoma (OSCC) is one of the most common malignancies arising in oral cavity. Remarkable advancement in reconstructive surgery and diagnostic modalities has improved the survival rate of OSCC patients. However, treatment failure for OSCC patients has lethal potential due to locoregional recurrence and distant metastasis. Unfortunately, advanced OSCC remains refractory and lethal in >50% of the cases (1,2).

Preoperative chemoradiotherapy has become an established part of the clinical management of locoregionally advanced operable OSCC in order to control locoregional disease (3–7). The beneficial effects of preoperative chemoradiotherapy include downstaging of the primary tumor, an increased resectability rate and the elimination of micrometastases. Kirita *et al* (7) demonstrated that preoperative cisplatin (CDDP)-based intravenous chemotherapy and concurrent radiotherapy resulted in a clinical tumor response of 92.8% and a good prognosis, with a 79.3% 5-year overall survival rate, in cases of resectable advanced OSCC. Several other studies have also demonstrated improved 5-year survival rates by using this treatment in patients with advanced OSCC (4–8). However, successful and satisfactory response to preoperative chemoradiotherapy is seldom achieved in all patients with advanced OSCC. It is thus crucial to elucidate the molecular mechanism of the differential chemosensitivity and identify molecular markers to distinguish between responders and non-responders (9–11).

It is well known that cancer cells are exacerbated by chronic inflammation (11). The primary role in this linkage is played by cytokines and chemokines produced by cancer cells as well as activated immune cells (12). Interleukin-6 (IL-6), a pleiotropic cytokine with a variety of biological activities, is secreted by different cell types including macrophages, T- and B-lymphocytes, fibroblast, endothelial cells, keratinocytes and cancer cells (13). Reportedly, an increased expression of IL-6 has been investigated in different types of cancers and high serum levels of IL-6 have been associated with metastasis and unfavorable prognosis (13–17). Furthermore, possible involvement of IL-6 signaling in the resistance to chemotherapy and radiotherapy has been documented in recent studies (18–20). In view of these findings, we hypothesized that IL-6 signaling pathway could be responsible for the progression and treatment resistance of OSCC. In the present study, we therefore examined the association of IL-6 expression with the clinical outcomes, especially with the histological response by preoperative chemoradiotherapy in patients with OSCC.

## Materials and methods

### Patient characteristics

We enrolled 78 patients with primary OSCC who were treated in the Department of Oral and Maxillofacial Surgery at Kyushu University Hospital from 2005 to 2011. The average age of the patients was 65.7±13.2 years (range, 19–89). Fifty-two patients were males and 26 were females. An informed consent was obtained from all the participant patients before any procedure or treatment was initiated (IRB no.: 25-227). Thirty-nine patients with locoregionally advanced OSCC underwent chemoradiotherapy preoperatively. Following the initial biopsy, all the specimens were fixed in 4% buffered formalin solution and were embedded in paraffin blocks. Subsequently, the paraffin-embedded specimens were processed to 5 *μ*m thick sections, stained with hematoxylin and eosin (H&E) and examined by experienced oral pathologists to confirm the diagnosis and histologic grade. The tumor stage was classified according to the TNM classification of the International Union Against Cancer (21). Tumor histologic grade was defined according to the WHO classification (22). The mode of the tumor invasion was determined from H&E stained specimens according to the Yamamoto-Kohama criteria as follows: grade 1 = well-defined borderline; grade 2 = cords, less-marked borderline; grade 3 = groups of cells, no distinct borderline; grade 4 = diffuse invasion (4C = cord-like type; 4D = widespread type) (23). Patients and tumor characteristics are shown in Table I.

### Preoperative chemoradiotherapy

The patients with locoregionally advanced OSCC received external beam irradiation to the primary tumor and the metastatic lymph nodes in daily fractions of 2 Gy, 5 times weekly, for 3 weeks. S-1 (TS-1; Taiho Pharmaceutical, Tokyo, Japan), an oral fluoropyrimidine preparation that consists of tegafur, 5-chloro-2,4-dihydroxypyridine (a dihydropyrimidine dehydrogenase inhibitor) and potassium oxonate which inhibits orotate phosphoribosyltransferase in the gastrointestinal tract, was used as an anticancer drug in this regimen. Oral administration of S-1 started one week prior to the radiotherapy and was continued throughout the radiotherapy period. Standard individual doses of S-1 were calculated according to the body surface area (BSA): BSA <1.25 m^2^, 80 mg; 1.25 ≤BSA <1.5 m^2^, 100 mg; BSA ≥1.5 m^2^, 120 mg. However, in patients with reduced renal function (decreased creatinine clearance values), S-1 was administered at a lower dose, generally one step lower than the standard dose. Radical surgery was carried out at 2–6 weeks (average, 27.5±5.22 days) after the end of the preoperative chemoradiotherapy.

### Immunohistochemistry

Immunohistochemical staining was performed on 5 *μ*m thick sections that were sliced serially from paraffin-embedded blocks after formalin fixation of the excised specimens. The sections were deparaffinized in xylene and rehydrated in graded ethanol (100, 95, 90, 85 and 75%). For antigen retrieval, the sections were immersed in Dako Target Retrieval Solution (Dako Cytomation, Denmark) and autoclaved at 121°C for 5 min. The endogenous peroxide activities were then eliminated with 3% hydrogen peroxide for 5 min, and the sections were rinsed twice for 10 min with phosphate-buffered saline (PBS) at pH 7.4. Non-specific protein bindings were attenuated by incubation for 30 min with 10% goat serum and then the sections were incubated with each primary antibody overnight at 4°C. The following antibodies were used: anti-human monoclonal IL-6 antibody (Abcam, UK; diluted 1:50), anti-human monoclonal IL-6 receptor (IL-6R) antibody (Invitrogen, CA, USA; diluted 1:50) and anti-human monoclonal phospho-signal tranducer and activator of transcription 3 (p-STAT3) antibody (Cell Signaling Technology, MA, USA; diluted 1:400). The sections were rinsed twice for 10 min with PBS and incubated with secondary antibody conjugated with peroxidase-labeled amino acid polymer for 1 h at room temperature. After being rinsed with PBS twice for 10 min, the immunoreactivity was visualized by immersing the sections in 3,3′-diaminobenzidine and 0.6% hydrogen peroxide (DAB substrate kit; Nichirei, Tokyo, Japan). Subsequently, the sections were counterstained with Mayer’s hematoxylin, dehydrated in graded ethanol (75, 85, 90, 95 and 100%), cleared with xylene and finally mounted with permanent mounting medium (Malinol mounting medium; Muto Pure Chemicals, Tokyo, Japan). Negative controls were prepared by substituting PBS for each primary antibody. To evaluate the expression of IL-6 in the OSCC, positively stained cancer cells were counted in at least three randomly selected areas at ×200 magnifications, and then each percentage of these positive cancer cells was calculated as labeling index (LI). The LI was computed by dividing the number of the positively stained cells by that of all the cancer cells. The patients with OSCC were divided into three groups as follows: negative group = LI <5%; low IL-6 group = 5% ≤ LI <30%; high IL-6 group = LI ≥30%.

### Clinicopathological evaluation of preoperative chemoradiotherapy

The classification of therapeutic efficacy established by Shimosato *et al* (24) was used to evaluate the histopathological response of the primary site tumors: grade 0, no noticeable change; grade I, minimal cellular changes, but the majority of the tumor cells appear viable; grade IIa, despite the presence of the cellular changes and partial destruction of the tumors, the tumor is still readily recognizable and many tumor cells appear viable; grade IIb, tumor destruction is extensive, but viable cell nests are present in small areas of the tumor (up to one quarter of the tumor mass, excluding areas of coagulative necrosis); grade III, only a few scattered, markedly altered and presumably non-viable tumor cells are present, singly or in small clusters, and few or no viable cells are seen; and grade IV, no tumor cells remaining in any section. Cutting of resected specimens was carried out by step-section method at intervals of 5 mm.

### Statistical analyses

All statistical analyses in the present study were performed by JMP software version 11 (SAS Institute, NC, USA). Chi-squared test was used to assess the significant differences between each group. Survival rates were also calculated by Kaplan-Meier method and the P-value was calculated by Log-rank test. A P-value of <0.05 was considered to be statistically significant.

## Results

### Expression of IL-6 proteins in the OSCC and adjacent nonmalignant oral mucosa

**Figure 1:**
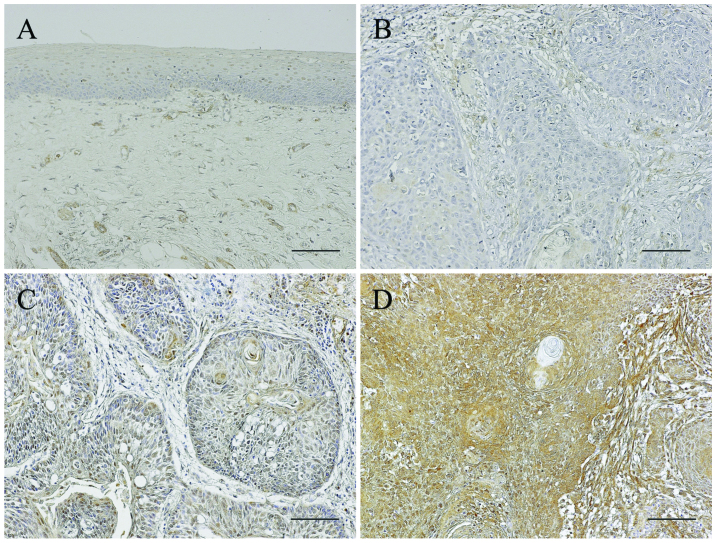
Immunoreactivity for IL-6 in the OSCC and adjacent non-malignant oral mucosa. Immunoreactivity for IL-6 is almost absent in the adjacent nonmalignant oral epithelia and weakly detected in the submucosal tissues (A). In the OSCC, the IL-6 expression pattern is different in the individual specimens. The patients are thus divided into three groups according to the IL-6 positive rates as follows: negative (b), low (C), and high expression groups (D). In the negative group, immunoreactivity for IL-6 is absent in most of the cancer cells, whereas stromal cells around the cancer nests express IL-6 slightly. IL-6 of the low expression group is scattered in the cancer nests whereas almost all the cancer cells express IL-6 in the high expression group. The stromal cells also strongly express IL-6 in the high expression group. Scale bars, 100 *μ*m. IL-6, interleukin-6; OSCC, oral squamous cell carcinoma.

Immunoreactivity for IL-6 was almost not detected in the adjacent non-malignant oral epithelia and weakly investigated in the submucosal tissues. In the OSCC patients, the IL-6 expression pattern was different in the individual specimens. The number of patients in the negative, low and high expression group were 24, 22 and 32, respectively. The OSCC patients were thus divided into three groups according to IL-6 positive rates as follows: negative, low and high expression groups. In the negative group, most cancer cells were negative for a IL-6 immunoreactivity whereas stromal cells around the cancer nests expressed IL-6 slightly. IL-6 expression of the low expression group was scattered in the cancer cells whereas almost all the cancer cells expressed IL-6 in the high expression group. The stromal cells also strongly expressed IL-6 in the high expression group (Fig. 1).

**Figure 2:**
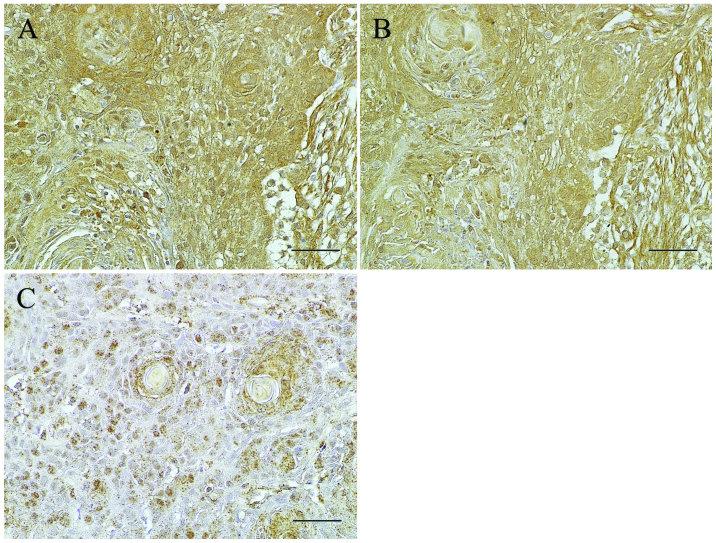
Expression of IL-6, IL-6R and p-STAT3 in OSCC. (A–C) IL-6, IL-6R and p-STAT3. In the IL-6 high expression group, IL-6R is detected in the cytoplasm and cytomembrane of almost all the cancer cells. p-STAT3 expression is localized in the nucleus of the cancer cells and scatters widely in the cancer nests. Scale bars, 50 *μ*m. IL-6, interleukin-6; OSCC oral squamous cell carcinoma; IL-6R, IL-6 receptor; p-STAT3, phospho-signal tranducer and activator of transcription 3.

In order to examine whether IL-6 signaling is activated in the OSCC cells, the expression of IL-6R and p-STAT3 was further examined immunohistochemically in the patients of the high IL-6 expression group. IL-6R was detected in the cytoplasm and plasma membrane of almost all the cancer cells and observed in harmony with that of IL-6 in almost all the OSCC cells of the IL-6 high expression group (Fig. 2A and B), but not or weakly in the negative and low expression group (data not shown). p-STAT3 expression was localized in the nucleus of the cancer cells and scattered widely in the cancer nests (Fig. 2C).

### Association of IL-6 expression with clinicopathologic characteristics in OSCC patients

The associations of IL-6 expression with the clinicopathologic factors of the OSCC patients were examined. The patients in the high expression group had a more advanced clinical stage than those in the negative and low expression groups. Furthermore, the prevalence of the cervical lymph node or distant metastasis in the high expression group was significantly higher than those in the negative and low expression groups (P<0.05, Chi-squared test). On the contrary, other clinical factors including gender, primary site, clinical T classification, local recurrence rate, histologic grade and mode of invasion did not show significant differences among these groups (Table I).

### Association of IL-6 expression with histopathologic tumor response to preoperative chemoradiotherapy for locally advanced OSCC

In the 39 patients who underwent preoperative chemoradiotherapy for locally advanced OSCC, the association of IL-6 expression in the cancer cells with histologic tumor response in the resected specimens was further examined. Equal to or higher than Grade IIb were judged to be histologically effective. All the patients in the negative group were good responders, whereas only a third of the patients had a histologically effective response in the high expression group. Increased IL-6 expression in the cancer cells was significantly associated with poor response to preoperative chemoradiotherapy (^*^P<0.05, ^**^P<0.01, Chi-squared test Table II).

### Expression of IL-6, IL-6R and p-STAT3 in the residual cancer cells after preoperative chemoradiotherapy

**Figure 3:**
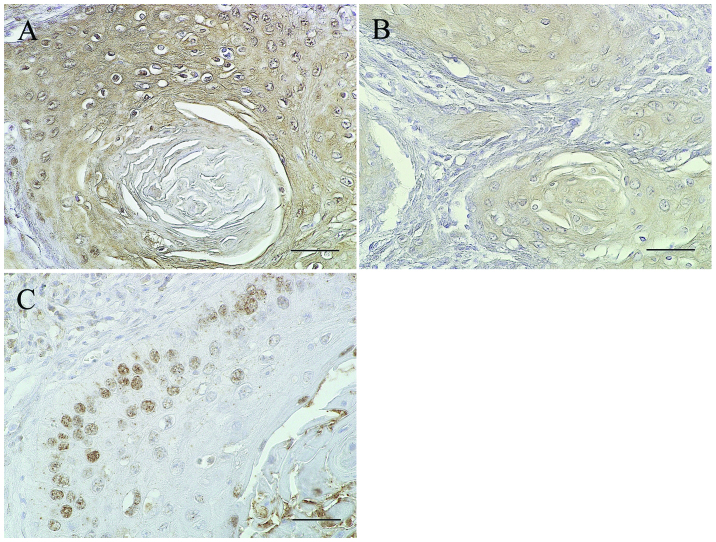
Expression of IL-6, IL-6R and p-STAT3 in the residual cancer cells after chemoradiotherapy. The expression of IL-6, IL-6R and p-STAT3 was examined immunohistochemically in the 12 patients of the high expression group with poor response to preoperative chemoradiotherapy. (A–C) In all the cases, IL-6 and IL-6R is detected in almost all the residual cancer cells, while p-STAT3 is mainly expressed in the outer layers of the cancer nest. Scale bars, 50 *μ*m. IL-6, interleukin-6; IL-6R, IL-6 receptor; p-STAT3, phospho-signal tranducer and activator of transcription 3.

In order to examine whether STAT3 signaling is also activated in the residual cancer cells after chemoradiotherapy, the expression of IL-6R and p-STAT3 was further examined immunohistochemically in the 12 patients of the high expression group with poor response to chemoradiotherapy. In all the cases, IL-6R was detected in the cytoplasm and cytomembrane of almost all residual cancer cells, while p-STAT3 was expressed only in the outer layers of the cancer nest (Fig. 3).

### Comparison of clinical outcomes and prognosis among the groups with differential immunoreactivities for IL-6

**Figure 4:**
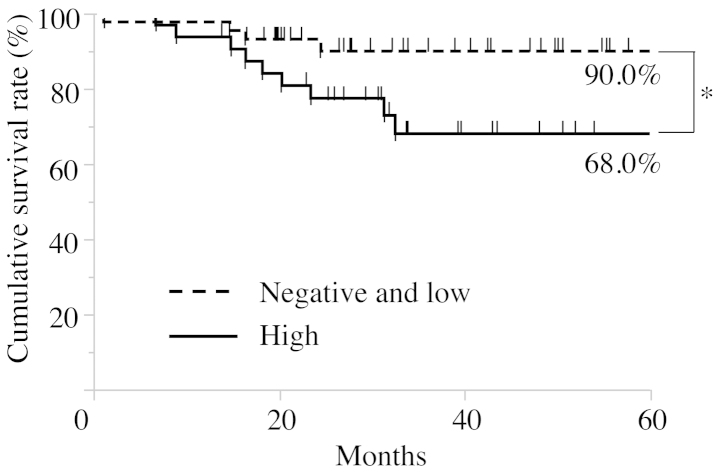
Survival curves according to immunoreactivity for IL-6 in OSCC. The survival rates are calculated by the Kaplan-Meier method. In the cause-specific cumulative survival curves, the patients in the high expression group have a significantly poorer outcome than those in the negative and low expression groups (Log-rank test, P<0.05). The cumulative survival rate for 5 years in the high expression group is 68.0%, whereas that in the negative and low expression groups is 90.0%. IL-6, interleukin-6; OSCC, oral squamous cell carcinoma.

For the purpose of evaluating the correlation between immunoreactivity for IL-6 in cancer cells and the clinical outcomes of the patients with OSCC, the survival rates were calculated by the Kaplan-Meier method. In the cause-specific cumulative survival curves, the patients in the high expression group had a significantly more unfavorable outcome than those in the negative and low expression groups (Log-rank test, P<0.05; Fig. 4). The cumulative survival rate for 5 years in the high expression group was 68.0%, whereas that in the negative and low expression groups was 90.0%.

## Discussion

In the present study, we examined the association of IL-6 expression in cancer cells with clinicopathological factors in the OSCC patients by immunostaining. There have been several studies on the correlation of the serum IL-6 concentration with the prognosis in patients with OSCC, but not with the expression of IL-6 proteins in the OSCC specimens (25–29). Immunostaining for IL-6 in OSCC revealed that the increased expression was more frequently observed in patients with an advanced clinical stage, cervical lymph node metastasis or distant metastasis. Shinriki *et al* (30), demonstrated by using tocilizumab, humanized anti-IL-6R antibody, that IL-6 signaling stimulated VEGF-C synthesis and lymphangiogenesis in OSCC. Recent studies revealed that elevated serum levels of IL-6 were significantly associated with the progression of malignancies, such as, ovarian cancer, esophageal cancer, and head and neck cancer (26,31,32). Furthermore, positive correlation between salivary IL-6 levels and locoregional recurrence have been observed in the OSCC patients (29), supporting our results, and suggesting that IL-6 plays a key role in the progression of OSCC.

Binding of IL-6 with its receptor initiates homodimerization of gp130 and then triggers signaling cascades through JAK-STAT, Ras-MAPK and PI3K-Akt pathways (13,33). Notably, constitutive activation of STAT3 has been reported to contribute to oncogenesis in a variety of malignancies (26,34–36). It is well known that IL-6 induces the transient phosphorylation of STAT3 and leads to its translocation into the nucleus. Expression of IL-6R and p-STAT3 was thus examined in the present study. The expression of IL-6R was observed in harmony with that of IL-6 in almost all the OSCC cells of the IL-6 high expression group, but not or weakly in the negative and low expression group. Furthermore, the expression of p-STAT3 was investigated in the nucleus of the cancer cells, suggesting that STAT3 signaling is activated through autocrine loop stimulation between IL-6 and IL-6R in OSCC cells.

Previous studies showed that IL-6 signaling pathway could be responsible for acquirement of malignant transformation, such as the ability to invade and metastasize, a migratory capacity, and resistance to chemotherapy or radiotherapy (18–20,37–39). Therefore, association of IL-6 expression with histopathologic tumor response to chemoradiotherapy was examined in the patients with locoregionally advanced OSCC treated by the preoperative chemoradiotherapy with S-1. The results showed that increased IL-6 expression in the cancer cells was significantly associated with poor response to preoperative chemoradiotherapy. Furthermore, the residual cancer cells in the resected specimens expressed IL-6, IL-6R and p-STAT3. Chen *et al* (18) demonstrated the significance of IL-6 signaling pathway in the resistance of pharyngeal cancer to irradiation and the epidermal growth factor receptor inhibitor. Sugimura *et al* (40) also showed that Let-7, one of the microRNAs, modulates the chemosensitivity to cisplatin through the regulation of IL-6/STAT3 signaling pathway in esophageal squamous cell carcinoma. In ovarian cancer, wang *et al* (41) reported that autocrine production of IL-6 confers resistance to cisplatin and paclitaxel. Gilbert *et al* (42) also demonstrated that IL-6 secreted from the endothelial cells after treatment with doxorubicin induced chemoresistant niche and is associated with increased resistance to DNA damaging agents in paracrine manner. Although the detail mechanism in OSCC remains to be elucidated in the present study, these results suggest that activation of IL-6/STAT3 signaling pathway through autocrine and paracrine stimulation may be involved in modulation of chemosensitivity to anticancer drugs. In addition, it was also suggested that IL-6 expression in cancer cells could be a useful predictive factor for tumor response to chemoradiotherapy in OSCC.

In the present study, we also examined the association of the IL-6 expression with the prognosis of patients with OSCC. In the cause specific cumulative survival curves, the patients in the high expression group had a significantly more unfavorable outcome than that in the negative and low expression groups. However, the association between the increased IL-6 expression and clinical prognosis for head and neck cancer patients remains controversial. Chen *et al* (43) showed that high IL-6 expression in tumor cells was significantly associated with poor prognosis in OSCC patients. Duffy *et al* (26) also demonstrated that pretreatment serum IL-6 levels could be a valuable biomarker for predicting recurrence and overall survival among HNSCC patients. These results were consistent with our results in the present study. On the contrary, Wang *et al* (44) reported that the patients with positive expression of IL-6 mRNA transcripts in cancer cells had a significantly favorable survival rate compared with those with the negative expression. This contradictory conclusion may have resulted from the use of different study populations or methodologies, or from different etiologies including smoking, alcohol consumption and betel nut chewing.

In conclusion, we showed that high expression of IL-6 in cancer cells predict poor response to chemoradiotherapy and unfavorable prognosis in patients with OSCC. Although further studies are needed to understand the complex roles of IL-6 signaling, completely revealing the functions of IL-6 during tumor progression will lead to new approaches for the treatment of OSCC.

## Figures and Tables

**Table I:** Association of IL-6 expression with clinical characteristics in OSCC.

Clinical factors	Cases (%)	IL-6 expression			P-value
		Negative	Low	High	
Gender					N.S.
Male	52 (66.7)	16	12	24	
Female	26 (33.3)	8	10	8	
Primary site					N.S.
Tongue	42 (53.9)	14	15	13	
Gingiva	26 (33.3)	7	5	14	
Buccal mucosa	7 (9.0)	2	1	4	
Oral floor	3 (3.8)	1	1	1	
Clinical stage					P<0.05
I and II	42 (53.8)	17	14	11	
III and IV	36 (46.2)	7	8	21	
Clinical T stage					N.S.
T1/T2	50 (64.1)	20	15	15	
T3/T4	28 (35.9)	4	7	17	
Nodal metastasis					P<0.05
Yes	35 (44.9)	7	7	21	
No	43 (55.1)	17	15	11	
Local recurrence					N.S.
Yes	14 (17.9)	3	2	9	
No	64 (82.1)	21	20	23	
Distant metastasis					P<0.05
Yes	5 (6.4)	0	0	5	
No	73 (93.6)	24	22	27	
Histologic grade					N.S.
Grade 1	29 (37.2)	7	9	13	
Grade 2	49 (62.8)	17	13	19	
Mode of invasion					N.S.
Grade 1/2/3	61 (78.2)	19	17	25	
Grade 4C/4D	17 (21.8)	5	5	7	

Values expressed as a number of patients. Chi-squared test are used to assess the significant differences between each group. N.S., not significant.

**Table II:** Association of IL-6 immunoreactivity with histopathologic tumor response to preoperative chemoradiotherapy.

IL-6 expression	Pathologic response	
	Effective (IIb-IV)	Non-effective (I-IIa)
Negative^a,b^	5	0
Low	11	5
High	6	12

Values expressed as a number of patientz. Chi-squared test was used to assess the significant differences between each group (^a^P<0.05, negative vs. low group; ^b^P<0.01, negative vs. high group).

## Abbreviations

IL-6: interleukin-6
OSCC: oral squamous cell carcinoma
p-STAT3: phospho-signal tranducer and activator of transcription 3
